# Microscale residual stresses in additively manufactured stainless steel

**DOI:** 10.1038/s41467-019-12265-8

**Published:** 2019-09-25

**Authors:** Wen Chen, Thomas Voisin, Yin Zhang, Jean-Baptiste Forien, Christopher M. Spadaccini, David L. McDowell, Ting Zhu, Y. Morris Wang

**Affiliations:** 1 Lawrence Livermore National Laboratory, Livermore, California 94550 USA; 2 Woodruff School of Mechanical Engineering, Georgia Institute of Technology, Atlanta, Georgia 30332 USA; 3 Present Address: Department of Mechanical and Industrial Engineering, University of Massachusetts, Amherst, Massachusetts 01003 USA

**Keywords:** Mechanical properties, Metals and alloys, Theory and computation

## Abstract

Additively manufactured (AM) metallic materials commonly possess substantial microscale internal stresses that manifest as intergranular and intragranular residual stresses. However, the impact of these residual stresses on the mechanical behaviour of AM materials remains unexplored. Here we combine in situ synchrotron X-ray diffraction experiments and computational modelling to quantify the lattice strains in different families of grains with specific orientations and associated intergranular residual stresses in an AM 316L stainless steel under uniaxial tension. We measure pronounced tension–compression asymmetries in yield strength and work hardening for as-printed stainless steel, and show they are associated with back stresses originating from heterogeneous dislocation distributions and resultant intragranular residual stresses. We further report that heat treatment relieves microscale residual stresses, thereby reducing the tension–compression asymmetries and altering work-hardening behaviour. This work establishes the mechanistic connections between the microscale residual stresses and mechanical behaviour of AM stainless steel.

## Abstract (web summary)

The impact of grain-scale residual stresses on the mechanical behaviour of 3D-printed metals and alloys remains unexplored. Here, the authors combine in situ synchrotron X-ray diffraction and computer simulations to link residual stresses in steel to its tensile behaviour.

## Introduction

Residual stress is one of the most critical issues for additively manufactured (AM) metallic materials^1–5^. Its presence can influence the mechanical behaviour of AM parts. This issue is especially significant for AM materials processed by selective laser melting (SLM), which inevitably results in substantial residual stresses. The buildup of high residual stresses during SLM processing can induce the damage and eventual failure of AM parts in service. Hence, mitigation of residual stresses is considered as one of the most outstanding challenges in the AM field. To palliate the deleterious effects of residual stresses, a fundamental understanding of their impact on the mechanical behaviour of AM materials is needed.

The residual stresses in polycrystalline materials can be categorized according to two major classes:^6,7^ macroscale and microscale residual stresses. The so-called Type I macroscale residual stresses are distributed across the overall dimension of a part and contribute to its distortion. For example, they arise due to long-range gradients of plastic deformation in the part. By contrast, the microscale residual stresses manifest at the scale of microstructure and are categorized into two types: Type II intergranular residual stresses and Type III intragranular residual stresses. Type II self-equilibrates over a length scale of grains and results from strain incompatibility between grains. Type III is associated with the heterogeneous microstructure such as dislocation cells inside grains and also satisfies the self-equilibrium condition. In general, microscale residual stresses develop after material processing and subsequently evolve under applied loading. Following the literature^8^, we use the terms ‘residual stress’ and ‘internal stress’ interchangeably with regard to these microscale residual stresses. To understand and control the microscale residual stresses, it is necessary to track their spatiotemporal evolution in real time at high resolution. This has been often pursued by means of in situ X-ray or neutron diffraction methods^8–17^.

The additive manufacturing of metallic materials via laser powder-bed-fusion (L-PBF) results in highly non-equilibrium microstructures with a high density of dislocations, irregular and tortuous grain morphologies, cellular structures and chemical segregation^1,4^. These microstructural characteristics can result in mechanical properties that significantly differ from materials produced by the conventional methods such as wrought and cast^5,18,19^. During L-PBF processing, the highly localized heating and rapid cooling of a melt pool, in conjunction with the layer-by-layer repetition of such a thermomechanical process, give rise to large thermal gradients and heterogeneous residual stresses within a non-equilibrium microstructure^20–23^. The thermal gradients are affected by many processing parameters^24^, including powder-bed temperature, laser power, powder thermophysical characteristics, melt pool size, etc. The convolution of these parameters often leads to a complex residual stress field. Although studies of the macroscale residual stresses in AM materials have revealed various deleterious effects such as loss of net shape, detachment from support structures or failure of the build parts^25^, the ways in which microscale residual stresses influence the mechanical performance of AM materials remain elusive. This is due to the difficulty in measurement and understanding of the spatiotemporal evolution of residual stresses at the scale of individual grains or phases^6^.

Here we present a combined experimental and modelling study of the microscale residual stresses in AM 316L austenitic stainless steel. We perform in situ synchrotron X-ray diffraction (SXRD) measurements of lattice strains in AM stainless steel under uniaxial tension. Micromechanics and crystal plasticity finite element (CPFE) models are developed to understand the impact of elastic anisotropy, progressive yielding and hardening on the extent and evolution of lattice strains and associated Type II intergranular residual stresses. We measure pronounced tension–compression asymmetries in yield strength and strain hardening for AM stainless steel. Combining the experimental and CPFE modelling results, we show that the tension–compression asymmetries are associated with back stresses originating from heterogeneous dislocation distributions and resultant Type III intragranular residual stresses. Our work not only demonstrates an effective approach to quantitatively evaluate the microscale residual stresses of both intergranular and intragranular characters, but also conveys practical ramification that the microscale residual stress effects should be carefully considered when using additive manufacturing to design and build complex components for structural applications.

## Results

### Microstructure

**Fig. 1:**
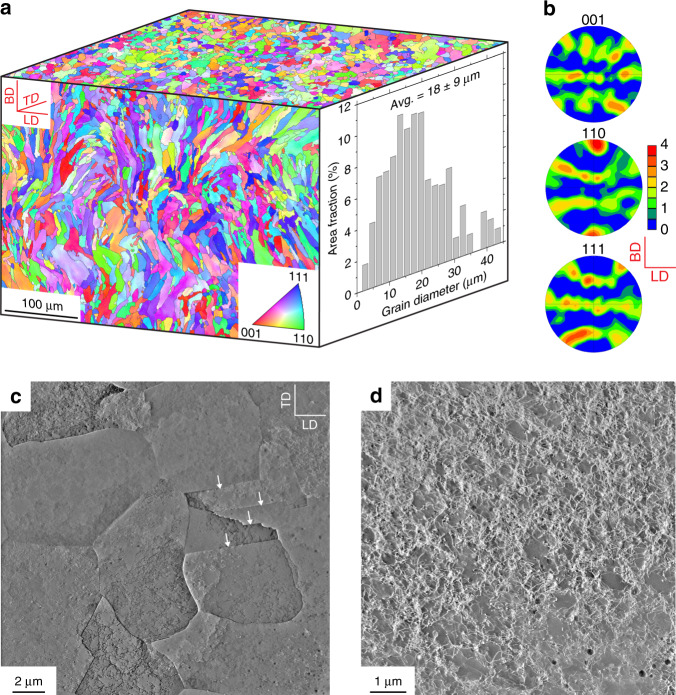
Microstructure of as-printed 316L stainless steel. **a** EBSD image along the build direction (BD) and transverse direction (TD), respectively. The EBSD image along the loading direction (LD) is similar to that along TD. The grain size distribution is obtained from the top surface (the TD-LD plane) in the image. **b** The 001, 110, 111 pole figures corresponding to the EBSD image taken along BD. **c** Top-view HAADF STEM image of the same sample in **a**. Tangled dislocations and a few twin boundaries (marked with white arrows) are visible; cellular structures are poorly defined. **d** A higher resolution HAADF STEM image of cellular structures compared to **c**. Some precipitates are visible

We used an open architecture Fraunhofer L37 L-PBF machine to build 316L stainless steel plates with the face-centred cubic structure. The detailed laser conditions are given in the Methods section and Supplementary Table 1. The electron backscatter diffraction (EBSD) image in Fig. 1a shows that grains in the as-printed sample are equiaxed from the top view and slightly elongated from the side view. The average grain size is 18 ± 9 µm, as measured from the top view. A rather weak texture is revealed in the build plane (Fig. 1b). The high-angle annular dark-field (HAADF) scanning transmission electron microscopy (STEM) images in Fig. 1c, d reveal negligible chemical segregation in the as-printed sample, as confirmed by elemental map shown in Supplementary Fig. 1. In the HAADF STEM image of Fig. 1d, the dislocation structures are not well defined, as they consist of tangled dislocations and are decorated with some visible precipitates. A low density of twin boundaries is observed inside grains. To study the effect of microscale residual stresses, samples were annealed at stress-relief conditions (500 °C, 4 h) and were used for comparison with as-printed samples (Methods and Supplementary Fig. 2). Overall, the 316L stainless steel samples built from the Fraunhofer machine in this study differ from those built with the commercial Concept machine reported previously^5^, as the latter possessed a relatively strong texture as well as significant chemical segregation in the solidification cell walls.

### In situ SXRD measurement

**Fig. 2:**
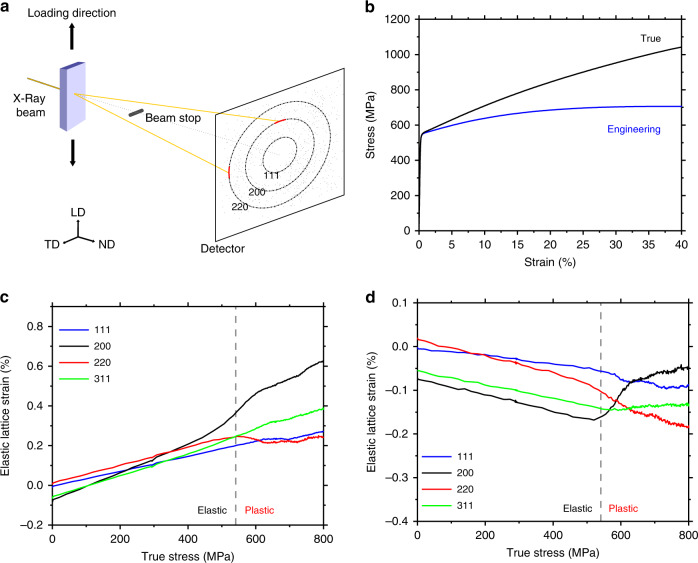
Lattice strain behaviour of an AM 316 stainless steel sample measured via in situ SXRD. **a** Schematic of in situ SXRD setup, where the loading direction (LD), transverse direction (TD) and normal direction (ND) are illustrated. **b** Engineering and true stress–strain curves of uniaxial tension, plotted up to the onset of necking. **c** In situ SXRD results of the lattice strain (*ɛ*^*hkl*^) along LD in four grain families of {111}, {200}, {220} and {311}, respectively, plotted against the macroscopic true stress of the sample. The 0.2% offset yield strength (*σ*_Y_) is marked with a dashed line, separating the elastic and plastic regimes. The average statistical uncertainty of the calculated strains is about 0.01%. **d** Same as **c**, except along TD

To investigate the microscale residual stresses in AM 316L stainless steel, we performed in situ SXRD measurements of lattice strains in an as-printed sample under uniaxial tension (Fig. 2a). Figure 2b shows the engineering and true stress–strain (*σ* – *ε*) curves up to the onset of necking, respectively. The 0.2% offset yield strength *σ*_Y_ is 541 ± 11 MPa, which is consistent with the earlier results^5,18^. Such high strength is two to three times those of coarse-grained counterparts and has been attributed to the printing-induced sub-grain microstructures such as dislocation cells^5^. Figure 2b also shows that further increase of the applied stress beyond *σ*_Y_ results in significant strain hardening, which is due to the deformation-induced evolution of heterogeneous microstructures^5^. For polycrystalline materials, the {*hkl*} grain family refers to a set of grains having the normal vector of {*hkl*} planes in a common direction. The lattice strain for the {*hkl*} grain family is defined as *ɛ*^*hkl*^ = (*d*^*hkl*^ − $d_0^{hkl}$)/$d_0^{hkl}$, where $d^{hkl}$ and $d_0^{hkl}$ denote the interplanar spacing of {*hkl*} planes under loading and at the stress-free state, respectively^11^. The stress-free lattice spacing $d_0^{hkl}$ was determined by annealing an as-printed sample at 1200 °C for 1 h. Figure 2c shows the lattice strain along the loading direction (LD) against the macroscopic true stress for four representative grain families of {220}, {111}, {200} and {311}. In addition, Fig. 2d shows the lattice strain along the transverse direction (TD) against the macroscopic true stress for four grain families of {220}, {111}, {200} and {311}. The lattice spacing as a function of the azimuthal angle can be seen in Supplementary Fig. 3. It is noteworthy that the constituent grains in the {*hkl*} grain family along LD are largely different from those in the {*hkl*} family along TD, and there is no clear relation between them^8^.

It is seen from Fig. 2c that prior to tensile loading, the {200} grain family exhibits the largest magnitude of residual lattice strain (being negative) along LD. In comparison, the {111} and {220} families show negligibly small residual lattice strains, whereas the {311} family exhibits a residual lattice strain similar to that of the {200} family. These residual lattice strains originate from the complex thermomechanical history associated with L-PBF processing. Figure 2c also shows that upon tensile loading, each of the four grain families exhibits a nearly linear increase of lattice strain against applied stress when the macroscopic tensile stress–strain response is within the linear elastic regime. This is followed by a nonlinear behaviour of lattice strain when the applied stress approaches and exceeds *σ*_Y_. More specifically, in the elastic regime, the {200} family shows the highest rate of increase of lattice strain, which indicates the softest response. In contrast, the {111} family exhibits the stiffest response. The lattice strains of the other two families fall in between these two limits. Such anisotropic lattice strain responses in different grain families can be quantitatively characterized in terms of the diffraction elastic constant *E*^*hkl*^, which is given by the slope of the lattice strain vs. applied stress curve for each {*hkl*} grain family^8^. The fitted values of *E*^*hkl*^ are listed in Table 1, which reflects the elastic anisotropy of austenitic stainless-steel single crystals and are consistent with the lattice strain measurements of stainless steel processed by conventional routes^8^. As the applied stress approaches *σ*_Y_ (indicated by the vertical dashed line in Fig. 2c), the {200} family shows a markedly nonlinear increase of lattice strain with the increase of applied stress, whereas the {220} family shows a nonlinear decrease of lattice strain. The nonlinear lattice strain responses of the {311} and {111} families are less pronounced. A similar nonlinear behaviour of lattice strain has been shown by the previous studies of stainless steel processed by conventional routes^8^. They are attributed to the elastic and plastic anisotropy in different grain families, which will be further analysed by our computational modelling in this paper.

**Table 1:** Diffraction elastic constants *E*^*hkl*^ (in GPa) in the {*hkl*} grain family of stainless steel

	{200}	{311}	{220}	{111}
*E*^*hkl*^ (Exp)	139.1 ± 1.1	179.6 ± 1.2	219.1 ± 1.6	264.1 ± 1.6
*E*^*hkl*^ (Micro)	147.3	183.7	210.7	245.9
*E*^*hkl*^ (CPFE)	158.6 ± 2.3	184.5 ± 1.1	209.7 ± 1.9	236.4 ± 2.3
*α*^*hkl*^ (Micro)	0.74	0.94	1.07	1.18
*β*^*hkl*^ (Micro)	0.25	0.37	0.38	0.34

Results of *E*^*hkl*^ are compared from the SXRD experiment (Exp), micromechanics (Micro) model and CPFE simulation. Also listed are the normalized tensile stress *α*^*hkl*^ and normalized maximum resolved shear stress *β*^*hkl*^ from the micromechanics (Micro) model. See Methods for the standard deviation definitions

Similar to LD, Fig. 2d indicates that prior to tensile loading, the {200} family exhibits the largest magnitude of residual lattice strain (being negative) along TD. As noted earlier, the constituent grains in the {*hkl*} family along TD are largely different from those in the {*hkl*} family along LD, such that the lattice strains in the nominally identical {*hkl*} grain families along LD and TD cannot be simply related. Nonetheless, due to the effect of Poisson’s contraction, an initial increase of tensile stress along LD results in a linear decrease of lattice strain along TD. Further loading gives rise to a nonlinear response of lattice strain along TD. Altogether, the in situ SXRD results in Fig. 2c, d reveal the lattice strains in several representative grain families prior to and during tensile loading. These lattice strains are elastic in nature and thus are proportional to Type II intergranular residual stresses, which will be quantitatively evaluated using micromechanics and CPFE models later in this paper. Quantifying these Type II intergranular residual stresses provides a basis of further study of Type III intragranular residual stresses in AM stainless steel.

### Tension–compression asymmetry

**Fig. 3:**
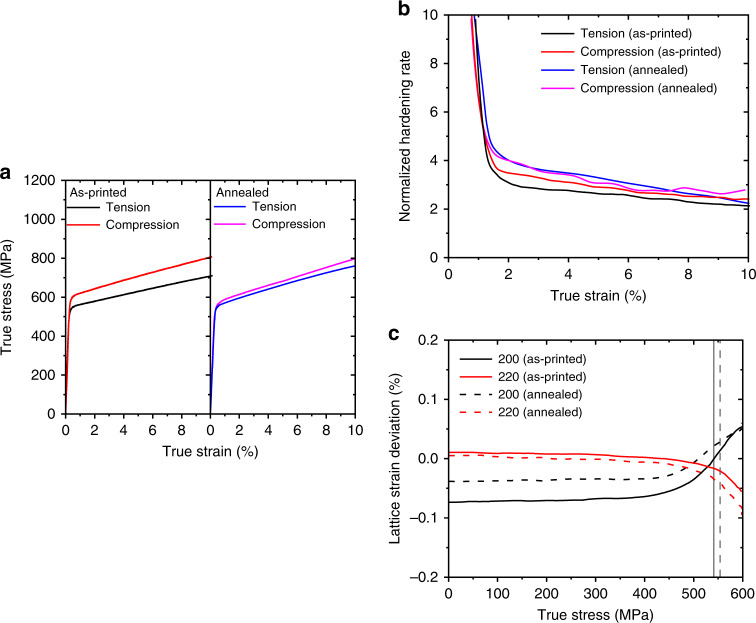
Experimental results of tension–compression asymmetry of AM 316L stainless steel. **a** True stress–strain curves of as-printed samples under uniaxial tension and compression. Also plotted are the corresponding results of annealed samples. **b** Normalized hardening rate vs. true strain corresponding to the four cases in **a**. **c** In situ SXRD results for as-printed and annealed samples under uniaxial tension, showing the lattice strain deviation (Δ*ε*^*hkl*^) as a function of applied true stress for {200} and {220} grain families

To investigate the impact of microscale residual stresses on the mechanical behaviour of AM stainless steel, we compare the stress–strain responses from uniaxial tension and compression experiments (Methods). As shown in Fig. 3a and Supplementary Fig. 4, the tensile and compressive stress–strain curves of as-printed samples exhibit pronounced asymmetries in yield strength and strain hardening. The 0.2% offset yield strength from uniaxial compression is 600 ± 13 MPa, which is higher than that from uniaxial tension 541 ± 11 MPa by ~60 MPa. In addition, the normalized strain hardening rate, (*dσ/dε*)*/σ*, is appreciably higher under compression than tension (Fig. 3b). For comparison purpose, we also measured the tensile and compressive stress–strain curves (Fig. 3a) for samples after stress-relief annealing at 500 °C for 4 h (Methods). In this case, the compressive yield strength decreases to 560 ± 14 MPa and the tensile yield strength increases to 554 ± 10 MPa. These results indicate that thermal annealing can significantly reduce the tension–compression asymmetry. This can be attributed to the relaxation of printing-induced non-equilibrium microstructures and associated microscale residual stresses in as-printed samples. If the asymmetry was primarily caused by printing-induced voids, the stress-relief annealing experiment at a moderate temperature would be unlikely to remove those voids and thus the tension–compression asymmetry would be little affected. In addition, as our AM samples have large grain sizes, the tension–compression asymmetry that is typically reported in nanocrystalline materials^26^ can be excluded. Despite the similar yield strengths in tension and compression after annealing, Fig. 3b shows that the strain hardening rate is still higher in compression than tension in annealed samples. Moreover, thermal annealing leads to an increased strain hardening rate in annealed samples than as-printed samples for both tension and compression beyond the initial yielding.

The in situ SXRD experiments were further conducted to compare the lattice strain responses between as-printed and annealed samples under uniaxial tension. Figure 3c shows the deviation of lattice strain *ε*^*hkl*^ from the linear response along LD, $\Delta \varepsilon ^{hkl} = \varepsilon ^{hkl} - \sigma _{\mathrm{a}}/E^{hkl}$, where *σ*_a_ is the applied tensile stress. Δ*ε*^*hkl*^ has often been used to examine the progressive yielding and hardening behaviour of different grain families. Figure 2c indicates that the {200} and {220} families exhibit more pronounced deviation from the linear response. Hence, Δ*ε*^200^ and Δ*ε*^200^ are used for further analysis of the lattice strain^8,9^ in the nonlinear regime against applied stress. Figure 3c indicates that deviation from the linear response occurs well below the 0.2% offset yield strength for both samples. In addition, the non-zero Δ*ε*^*hkl*^ prior to loading of the as-printed sample shows a marked initial value of compressive lattice strain, particularly for the {200} family, and thus suggests the high magnitude of Type II residual stresses in the as-printed sample. After annealing, such compressive shift reduces substantially but is not completely removed (Fig. 3c). The {220} family also demonstrates a qualitatively similar trend despite a much smaller initial value of tensile lattice strain prior to loading. These results suggest that moderate-temperature annealing can relieve but not fully eliminate the microscale residual stresses.

### Back-stress measurement

The pronounced tension and compression asymmetries of the as-printed samples suggest the development of substantial back stresses during L-PBF processing for stainless steel. The back stress is directional and is usually associated with the asymmetric tension and compression responses^14,27–30^. For example, in a material containing heterogeneous dislocation cell structures, long-range internal stresses (i.e., Type III residual stresses) develop with forward stresses in the hard regions of cell walls and back stresses in the soft regions of cell interiors^31,32^. As a result, back stresses resist the forward motion of dislocations and assist their reverse glide in the cell interiors, leading to a lower yield stress during reverse loading. This is known as the Bauschinger effect that gives rise to kinematic hardening^14,27–30^. The back stresses are expected to prevail in AM materials with heterogeneous microstructures. Using Dickson’s method^33^, we measured the effective back stress of *σ*_b_ ≈ 229 MPa at 3% strain under compressive loading–unloading and *σ*_b_ ≈ 180 MPa at 3% strain under tensile loading–unloading, respectively (Methods and Supplementary Fig. 5). These back stresses represent a significant portion of the tensile and compressive yield strengths of AM strainless steel, reflecting a strong Bauschinger effect. More importantly, the difference in the measured back stresses by ~50 MPa between tension and compression is close to the difference in the tensile and compresive yield strengths of ~60 MPa. This observation will be further examined using CPFE simulations to unravel the effects of back stresses originating from L-PBF processing and mechanical loading.

### Micromechanics modelling

The above in situ SXRD results have revealed the orientation dependence of lattice strain response that can be characterized by the diffraction elastic constant of each grain family^8^. The in situ SXRD results have also shown that different grain families begin to exhibit a nonlinear lattice strain response at different applied stresses. This progressive yielding behaviour can be understood by considering the maximum resolved shear stress or, equivalently, the maximum Schmid factor in each grain family. In this work, we develop a micromechanics model that provides an analytic solution of the diffraction elastic constant and Schmid factor in different grain families. These micromechanics results facilitate our understanding of how the lattice strain and progressive yielding depend on the elastic anisotropy of individual grain families. They are also used to benchmark CPFE simulations for further studies of the nonlinear lattice strain responses as well as the tension–compression asymmetries in AM stainless steel.

**Fig. 4:**
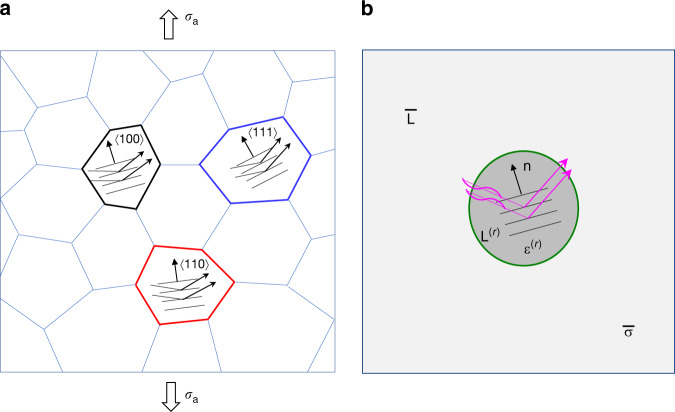
Schematic illustration of the micromechanics model of a polycrystalline aggregate. **a** Polycrystal consisting of different {*hkl*} grain families oriented along the uniaxial loading direction. For example, the {111} grain family refers to a set of grains with the unit normal vector **n** of {111} planes along the loading direction. X-rays reflected by the {*hkl*} planes are collected to track the average interplanar spacing of these {*hkl*} planes with deformation due to applied stress *σ*_a_. **b** In the self-consistent polycrystal model, each single-crystal grain is approximated as a spherical inclusion with the anisotropic elastic stiffness tensor **L**^(*r*)^ embedded in a homogeneous matrix with the effective stiffness tensor ${\bar{\mathbf{{L}}}}$

The micromechanics model illustrated in Fig. 4a is concerned only with the linear elastic response of a polycrystalline aggregate. The residual stress is not included in the grains, as the diffraction elastic constant is known to be independent of residual stress^8^. For each grain, its anisotropic elastic stiffness tensor is denoted as **L**^(*r*)^, where *r* represents a family of grains with the same crystallographic orientation. As shown in Fig. 4a, a representative volume element (RVE) is used to represent an infinite homogeneous matrix. Given the assumed random distribution of grain orientations, this RVE has an effective isotropic elastic response that is characterized by the isotropic elastic stiffness tensor ${\bar{\bf{L}}}$. As shown in Fig. 4b, a spherical inclusion is considered as a representative grain embedded in the RVE under the macroscopic stress $\bar{\boldsymbol{\sigma}}$. According to the Eshelby inclusion solution, the stress **σ**^(*r*)^ and strain **ε**^(*r*)^ in the spherical inclusion are uniform^34^. The self-consistent micromechanics method^34^ is used to determine the effective moduli of polycrystalline stainless steel. These results are combined to derive the analytic formulas of stress and strain in the {*hkl*} grain family (Methods).

To understand the lattice strain and stress responses during in situ SXRD experiments, we consider an applied uniaxial tensile stress *σ*_a_ and use the above micromechanics solution to derive the corresponding lattice strain *ε*^*hkl*^ and stress *σ*^*hkl*^ along LD in the {*hkl*} grain family in the linear elastic regime (Methods). It follows that the diffraction elastic constant is given by ${\it{{E}}}^{{\it{hkl}}}{\it{ = \sigma }}_{\mathrm{{a}}}/{\it{\varepsilon }}^{{\it{hkl}}}$. We also calculate the normalized tensile stress $\alpha ^{{\it{hkl}}} = \sigma ^{{\it{hkl}}}/\sigma _{\mathrm{a}}$, so as to compare the tensile stress in different grain families along LD, as well as the normalized maximum resolved shear stress $\beta ^{{\it{hkl}}} = \tau ^{{\it{hkl}}}/\sigma _{\mathrm{a}}$ among the 12 $\{111 \} \langle 110 \rangle$ slip systems, so as to determine the sequence of onset of plastic yielding in different grain families. The calculated *E*^*hkl*^, *α*^*hkl*^ and *β*^*hkl*^ for the four grain families are listed in Table 1. The diffraction elastic constants *E*^*hkl*^ from the micromechanics model confirm that the {200} and {111} families are the softest and stiffest along LD, respectively, which are consistent with the SXRD results and closely match the modelling results by Clausen et al.^8^. The relative magnitudes of the tensile stress along LD (given by *α*^*hkl*^) follow monotonically those of the diffraction elastic constant. More interestingly, the relative magnitudes of the maximum resolved shear stress (given by *β*^*hkl*^) and thus the maximum Schmid factor do not follow monotonically those of the diffraction elastic constant and tensile stress along LD. Due to the favoured orientation between the most stressed $\{111 \} \langle 110 \rangle$ slip plane and LD, the {220} family has the maximum resolved shear stress and thus the maximum Schmid factor, which implies the earliest onset of plastic yielding under uniaxial tension. The *β*^*hkl*^ values of both the {311} and {111} families are lower than that of the {220} family, indicating that yielding for these two families occurs at higher applied stresses than the {220} family. The {200} family still has the minimum resolved shear stress and thus the minimum Schmid factor, which indicates the latest onset of plastic yielding among the four grain families. Finally, we note that although the diffraction elastic constants are independent of the printing-induced residual stresses that exist prior to loading, the stress and yielding responses in each grain family can be affected by these residual stresses, which will be further analysed by CPFE modelling.

### CPFE modelling of nonlinear lattice strains

We develop a CPFE model (Methods) to study the nonlinear lattice strain behaviour as well as the impact of Type II and Type III residual stresses on the mechanical behaviour of AM stainless steel. The crystal plasticity constitutive equations are formulated within the rate-dependent, finite-strain framework of elastic–plastic deformation for individual grain crystals^35^. To account for the effects of Type II and Type III residual stresses, we introduce the eigen-strain tensor E^*^ and the back-stress tensor **B** in the crystal plasticity model, respectively. More specifically, E^*^ represents the printing-induced residual lattice strains and thus reflects the impact of Type II residual stresses. The components of E^*^ for different grain families were estimated based on in situ SXRD measurements before loading. On the other hand, the back-stress tensor **B** represents the effective internal stresses within grains arising from heterogeneous dislocation distributions, thus reflecting the impact of Type III residual stresses^14^. As such, the so-called intergranular and intragranular back stresses in previous studies^27,28^ are collectively considered as the Type III residual stresses in this work. The back-stress tensor **B** is assumed to be deviatoric within the present pressure-independent crystal plasticity model. The initial values of the back-stress tensor, denoted as **B**_0_, were assigned to represent the internal stresses arising from printing-induced heterogeneous dislocation structures. These initial values are responsible for the tension–compression asymmetry of yield strength of the as-printed samples. Furthermore, the back-stress tensor **B** in individual grains evolves with the local plastic shear on different $\{111 \} \langle 110 \rangle$ slip systems in a nonlinear manner with applied load. Such nonlinear response reflects the rapid development of back stresses as measured with ~ 200 MPa at 3% strain under uniaxial tension and compression, which indicates a significant impact of deformation-induced back stresses on the plastic responses of AM stainless steel during loading. We implemented the crystal plasticity model in the finite element simulation package ABAQUS/Explicit^36^ by writing a user material subroutine VUMAT. The finite element simulations generated the macroscopic stress–strain curves and lattice strain responses in different grain families (Methods).

**Fig. 5:**
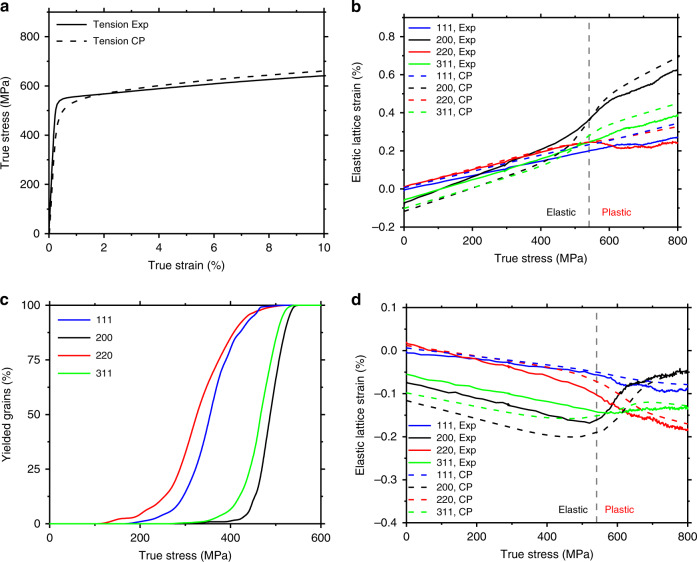
CPFE simulation results of stress and strain responses in as-printed stainless steel under uniaxial tension. **a** Comparison of the true stress–strain curves of uniaxial tension from experiment (Exp) and crystal plasticity (CP) finite element simulatoin. **b** Comparison of the lattice strains along LD against the macroscopic true stress for the {111}, {200}, {220} and {311} grain families from experiment and CPFE simulation. **c** Simulated volume fraction of plastically yielded grains within each family against the macroscopic true stress in the as-printed sample, showing progressive plastic yielding among different grain families. **d** Same as **b**, except along TD

Figure 5a shows the true stress–strain curve from CPFE simulation of uniaxial tension, which closely matches the experimental result. Figure 5b shows the simulated lattice strains along LD for the four grain families of {220}, {111}, {200} and {311} against the macroscopic tensile stress. The main features of the simulated lattice strains are all in accordance with the experimental results. The fitted values of diffraction elastic constants *E*^*hkl*^ from CPFE simulations (as listed in Table 1) are close to the SXRD and micromechanics results. Further parametric studies indicate that the residual lattice strains prior to loading are directly correlated to the eigen-strain tensor E^*^ and thus are responsible for Type II intergranular residual stresses.

To reveal the progressive yielding behaviour of different grain families and associated nonlinear lattice strain responses, Fig. 5c shows the volume fraction of plastically yielded grains against the macroscopic tensile stress for the four grain families. Suppose the mean yield stress for each grain family is given by the macroscopic stress giving 50% yielded grains. It is seen from Fig. 5c that the {220} family first yields, whereas the {200} family last yields; the mean yield stress of the {111} family is similar to {220} and the {311} family is similar to {200}. This sequence of progressive yielding is mostly consistent with the micromechanics model prediction, i.e., the relative magnitudes of *β*^*hkl*^ in Table 1. The only exception is the {311} family. This can be attributed to the large residual lattice strain in this grain family in the as-printed sample (Fig. 5b), which is accounted for in the CPFE model but not in the micromechanics model. In addition, local deformation incompatibilities between neighbouring grains can affect the stress state in individual grains, leading to the statistical variation of yield stresses of individual grains in each grain family and thus to the gradual increase of volume fraction of yielded grains for each grain family in Fig. 5c. Comparison of Fig. 5b, c also reveals that the highly nonlinear response of lattice strain vs. applied stress for the {200} family begins at a macroscopic stress much lower than the mean yield stress of this grain family. This indicates that the nonlinear lattice strain evolution for the {200} family is, in fact, initially associated with elastic deformation. That is, such nonlinearity arises due to stress redistribution to this softest grain family (i.e., with the lowest diffraction elastic constant shown in Table 1). As other grain families progressively yield, they shed their loads onto the grains in the {200} family that remains elastic.

In addition, Fig. 5d shows the lattice strains along TD. It is seen that the simulated residual lattice strain responses are consistent with the experimental results. The nonlinear lattice strains along TD in different grain families have similar origins as those along LD. However, the linear elastic lattice strains along TD are more complicated. For example, the non-monotonic variation of lattice strain in the {200} family around the macroscopic yield stress suggests the highly nonlinear interactions between this grain family and other families during load shedding and redistribution as other grain families become progressively yielded.

### CPFE modelling of tension–compression asymmetry

**Fig. 6:**
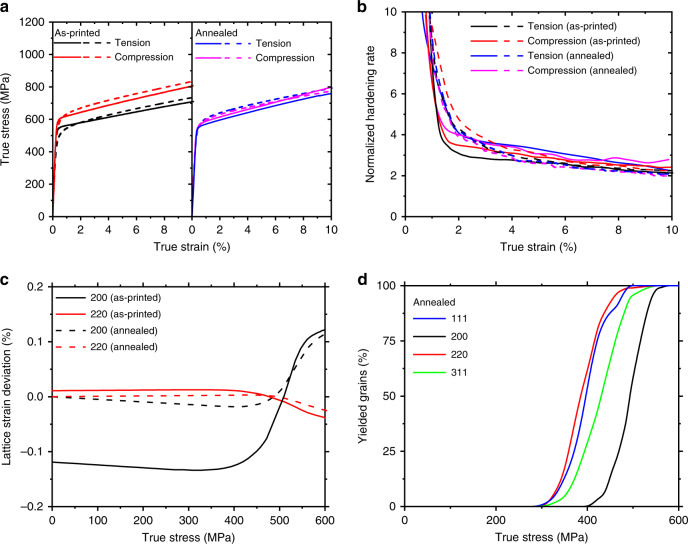
CPFE results of tension–compression asymmetry of AM stainless steel. **a** Comparison of true stress–strain curves under uniaxial tension and compression from experiment (solid lines) and simulation (dashed lines) for as-printed and annealed samples. **b** Comparison of normalized strain hardening rate vs. true strain under uniaxial tension and compression from the experiment (solid lines) and simulation (dashed lines) for as-printed and annealed samples. **c** Simulated lattice strain deviation (Δ*ε*^*hkl*^) as a function of applied stress for {200} and {220} grain families for as-printed and annealed samples under uniaxial tension. **d** Simulated volume fraction of plastically yielded grains within each family against the macroscopic true stress in the annealed sample, showing progressive plastic yielding among different grain families

We compare the CPFE simulations of uniaxial tension and compression to reveal the effects of microscale residual stresses on the tension–compression asymmetry of AM stainless steel. Figure 6a shows that the simulated tensile and compressive stress–strain curves of the as-printed sample agree with the corresponding experimental data. Further parametric studies indicate that the tension–compression asymmetry of yield strength is predominantly controlled by the initial values of the back-stress tensor **B**_0_. As described in the Methods section, we assigned identical values to **B**_0_ for all grains in the simulated polycrystalline aggregate, which approximately represent the average effect of type III intragranular internal stresses in these grains. That is, to match the simulation results of asymmetric tensile and compressive yield strengths with experimental ones, the initial back-stress components along LD and TD within the build plane are both chosen to be 30 MPa, respectively; the initial back-stress component along the build direction is chosen to be −60 MPa, so as to make **B**_0_ deviatoric. The shear components of **B**_0_ are chosen to be zero. The nonlinear relation between back stress and plastic strain adopted in the CPFE model is also important. This is because the deformation-induced back stresses increase quickly with applied stress, reaching ~200 MPa at the yield point for both tension and compression. As such, the back-stress component along LD in tension and compression at the respective yield point in CPFE simulations match the corresponding experimental data. These results demonstrate the different effects of printing-induced and deformation-induced back stresses. Nonetheless, as the back stresses represent the effective long-range resistances to dislocation glide within grains, the CPFE simulation results indicate that the asymmetry of tensile and compressive yield strengths in as-printed samples is caused primarily by the printing-induced back stress and associated Type III intragranular residual stresses, which arise from heterogeneous dislocation structures in as-printed samples. In addition, the asymmetric strain hardening rate is also captured in CPFE simulations (Fig. 6b) by fitting the constitutive parameters in the nonlinear relation of back stress vs. plastic shear strain. This indicates that both the printing- and deformation-induced microstructures and associated Type III intragranular residual stress affect the asymmetric evolution of strain hardening rate.

For comparison, Fig. 6a, b also show the simulated stress–strain curves and strain hardening rate–true stress curves of the annealed sample by taking the eigen-strain tensor E^*^ and the initial values of the back-stress tensor **B**_0_ as zero, while keeping other model parameters unchanged. It is seen that the tension–compression asymmetries in yield strength and strain hardening are completely removed in CPFE simulations. Parametric studies indicate that zeroing **B**_0_ is essential to remove the tension–compression asymmetries. Figure 6c shows the simulated lattice strain deviation Δ*ε*^*hkl*^ from the linear response for {200} and {220} grain families in both the as-printed and annealed samples under uniaxial tension. Their trends are similar to the correponding experimental results in Fig. 2c. However, the simulated initial values of Δ*ε*^*hkl*^ vanish and the nonlinearity of lattice strain reduces during loading. These responses arise from zeroing E^*^. Altogether, the CPFE results suggest that thermal annealing during experiment did not fully relax printing-induced heterogeneous microstructures for completely removing back stresses and residual lattice strains in as-printed samples. Finally, Fig. 6d shows the simulated volume fraction of plastically yielded grains within the four grain families against the macroscopic tensile stress in the annealed sample. Compared with the corresponding simulation results of the as-printed sample in Fig. 5c, the sequences of progressive yielding in different grain families are consistent between the two cases. However, the mean yield stressses of the {311} and {200} families in the annealed sample are remarkably reduced due to the absence of the residual lattice strains (by zeroing E^*^) in these two grain families. These results reinforce the notion that a stress-relief heat treatment of AM samples can alter their microscale residual stresses.

In summary, our work demonstrates that microscale residual stresses have profound impacts on the yielding and strain-hardening behaviour of AM stainless steel. The in situ SXRD experiments provide a powerful approach to unravel the residual lattice strains and associated Type II intergranular residual stresses for individual grain families in as-printed stainless steel as well as their evolution under applied loads. The combined SXRD and modelling results elucidate the effects of elastic anisotropy, progressive yielding and strain hardening on the extent and evolution of lattice strains and associated Type II intergranular residual stresses in different grain families. A pronounced tension–compression asymmetry of yield strength is observed, together with an asymmetric work-hardening behaviour. Such tension–compression asymmetries are shown to be governed by Type III intragranular residual stresses and associated back stresses, which arise from heterogeneous dislocation distributions that can be strongly influenced by both L-PBF processing and mechanical loading. Hence, it is important to distinguish the printing and loading-induced back stresses. Our CPFE simulations show that the former dictates the tension–compression asymmetry of yield strength in as-printed samples, whereas the latter can quickly build up during loading and thereby affect both the yield strength and strain-hardening responses. Both L-PBF processing and subsequent mechanical loading can contribute substantially to the back-stress evolution as measured from the loading–unloading experiment on as-printed samples, leading to the strong Bauschinger effect of AM stainless steel. Moreover, we show that thermal annealing of as-printed samples could mitigate both Type II and Type III residual stresses but is difficult to erase completely these microscale residual stresses. Altogether, our results demonstrate the quantitative and mechanistic connections between the microscale residual stresses and mechanical behaviour of AM stainless steel. Future studies on linking the printing parameters with the resultant microstructural heterogeneities and associated microscale residual stresses are necessary to enable the control and mitigation of these residual stresses. We expect that our work has general implications to AM metallic materials, as multiscale residual stress is a critical issue for this rapidly developing technology.

## Methods

### Materials processing

The 316L stainless-steel samples used in this study were fabricated by an open architecture Fraunhofer L37 laser powder-bed fusion machine equipped with a fibre laser of 400 W with a laser spot size of 207 μm (ellipse beam). The plasma atomized 316L powder was used (LPW Technology, UK). The powder has particle sizes ranging from ~20–80 μm with the mean value of ~ 30 μm, as described in our prior work^5^. Samples with an overall dimension of 40 mm × 40 mm × 3 mm were built on a 304 stainless steel substrate under the processing conditions listed in Supplementary Table 1. These parameters were selected based on a series of prior control experiments, so as to optimize the processing parameters and obtain near fully dense samples^37^.

### Microstructure characterization

To examine the microstructure of as-printed 316L stainless steel, EBSD tests were performed in an FEI Quanta 200 environmental scanning electron microscope equipped with a Hikary backscatter electron detector from EDAX at an accelerating voltage of 30 KeV. A step size of 0.1–2 μm was used depending on the magnification selected. EBSD samples were hand polished with abrasive papers of grits up to 5 µm, followed by polishing with diamond suspensions up to 1 µm and a final vibratory polishing for several hours. EBSD data were analysed with the Orientation Imaging Microscopy Analysis™ software provided by EDAX. To calculate the grain size, each grain was considered as a sphere from which the diameter (grain size) was deducted. Interfaces between grains were considered as low-angle grain boundary when the misorientation angle *θ* was 2–10° and high-angle grain boundary when *θ* > 10°.

### Transmission electron microscopy

TEM was conducted with an FEI Titan at 300 KeV. Bright-field and dark-field images were obtained in the TEM mode, and HAADF images were obtained in the scanning mode (STEM). TEM samples were mechanically polished down to a thickness near 100 µm with abrasive papers of grits up to 5 µm and with diamond suspensions up to 1 µm, and then electropolished with a 10% perchloric acid and 90% acetic acid solution at 5 °C. A twinjet Fischione polisher was used with the electrical voltage/current maintained at 30 V/50 mA.

### High-energy SXRD

SXRD experiment was conducted in situ during tensile testing with the Advanced Photon Source of Argonne National Laboratory at the beamline 6-ID-D. A high X-ray energy of 100 keV (*λ* = 0.123984 Å) was used. The X-ray beam size was 0.4 × 0.8 mm^2^ (height × width). Diffraction patterns were collected in transmission, every 2 s upon testing, on a GE amorphous silicon detector with 200 μm × 200 μm pixel size positioned 115.663 cm behind the sample. The samples were loaded with a Zwick-Roell Z2.5 tester equipped with a non-contact laser extensometer to measure the strain at a nominal strain rate of 5 × 10^–4^ s^−1^. The diffraction patterns were integrated in 10° azimuthal bins using XRDUA to obtain one-dimensional (1-D) diffraction patterns as a function of the azimuthal angle. To quantify the lattice strain, inhomogeneous strain (peak broadening) and texture changes during loading, the 1-D patterns were fit using a pseudo-Voigt function by a custom Matlab programme. To determine the stress-free lattice spacing $d_0^{hkl}$, the stress-free 316L reference sample was obtained by annealing an as-printed sample at 1200 °C for 1 h, followed by furnace cooling under argon environment. The stress-free sample is characterized by grain-orientation-independent diffraction peak positions. The sample geometry and testing conditions of the reference sample are the same as those of the as-printed and stress-released samples. As the measured lattice parameters are sensitive to the sample position, we positioned each sample against a reference that was not moved between tests. The following formula was used to calculate the error in *d*-spacing $\Delta d = \frac{{\lambda \times \cos \theta }}{{2 \times {\mathrm{sin}}^2\theta }} \times \frac{D}{{2 \times (L^2 + \, D^2)}} \times \Delta L$, where Δ*d* is the variation in *d*-spacing (mm), *λ* the X-ray wavelength (1.24 × 10^−8^ mm), *θ* is the scattering angle (°), *D* is the radial distance of the scattering angle on the detector (mm), *L* is the sample-to-detector distance (1155.56 mm), and Δ*L* is the estimated variation in sample-to-detector distance (<0.2 mm). The position of all samples in our experiments is determined by the tensile machine grips, which is fixed against camera. The major error bar comes from the sample thickness variation. Standard deviations for the experimental elastic moduli for each grain family shown in Table 1 were determined from the linear regression analysis.

### Mechanical testing

The built plates were removed from the substrate by electrical discharge machining (EDM). Dog-bone-shaped uniaxial tension samples with gauge dimension of 6.5 mm (length) × 1.4 mm (width) × 1.4 mm (thickness) and compression pillars of 1.5 mm × 1.5 mm × 3 mm (height) were cut from the centre of the plate by EDM, respectively. To ensure the similar thermal history and microstructure, tension and compression samples were cut from neighbouring area along the same direction. Mechanical tests were carried out via an Instron (Norwood, MA, USA) 3345 universal test machine at a nominal strain rate of 5 × 10^−4^ s^−1^. The strain was measured by an Instron non-contact AVE2 video extensometer (Norwood, MA, USA) with a displacement resolution of 1 μm. Before tests, all samples were polished down to metallurgical grit of 1200. Apiezon grease was applied at the two specimen/platen interfaces to reduce interfacial friction during loading. To study the influence of residual stresses on the yield asymmetry, both as-printed and annealed samples were tested. The samples were annealed at 500 °C for 4 h in a tube furnace at a heating rate of 25 °C/min under protective argon atmosphere and subsequently furnace cooled. The temperature was selected based on a series of hardness measurements by which the original microstructure was almost retained, while residual stresses could be significantly reduced (Supplementary Fig. 2 and Fig. 6). An MTS Nanoindenter XP system equipped with a Berkovich-type diamond tip was applied to probe the hardness of the as-printed and annealed samples under a strain rate of 5 × 10^−3^ s^−1^. An indentation depth of 2 μm was implemented. To minimize the surface toughness effect, all nanoindentation samples were polished using 1 µm diamond suspension. For each experimental condition, ten indents spaced more than 30 µm apart were recorded. The back stress, *σ*_b_, within the as-printed sample was measured through loading and unloading up to a strain of ~3% based on Dickson’s method^33^ (Supplementary Fig. 5). The unloading stress, *σ*_u_, was obtained by fitting the unloading curve through a straight line representing the linear elastic response during unloading.

### Micromechanics model

To predict the diffraction elastic constant, we developed a micromechanics model of a polycrystalline aggregate based on the self-consistent model and Eshelby inclusion solution^34^. Kröner has previously derived a self-consistent solution of the diffraction elastic constant^38^. However, his solution was published in German and not easily accessible. Here we adopted the modern micromechanics notation to derive the analytic solution of diffraction elastic constant under uniaxial loading. As illustrated in Fig. 4b, an RVE is used to represent an infinite homogeneous matrix with the effective isotropic elastic stiffness tensor ${\bar{\bf{L}}}$. A representative single-crystal grain with the elastic stiffness tensor **L**^(*r*)^ is approximated as a spherical inclusion embedded in a homogeneous matrix subjected to the macroscopic applied stress tensor ${\bar{\boldsymbol{\sigma }}}$. The stress tensor **σ**^(*r*)^ and strain tensor **ε**^(*r*)^ within the grain inclusion can be derived based on the self-consistent method and the classical Eshelby solution^34^, provided the polycrystal response follows linear elasticity. For a polycrystal under an applied uniaxial tensile stress *σ*_a_, the corresponding macroscopic stress tensor can be written as $\bar \sigma _{ij} = \sigma _{\mathrm{a}}n_in_j$. Here, *n*_*i*_ denotes the unit vector along LD, which is given by $(n_1,n_2,n_3) = (h,k,l)\sqrt {h^2 + k^2 + l^2}$ when the {*hkl*} grain family is along LD. In each grain inclusion, the lattice strain *ε*^*hkl*^ along *n*_*i*_ can be calculated as ${\it{\varepsilon }}^{{\it{hkl}}} = \varepsilon _{ij}^{(r)}n_in_j$, where $\varepsilon _{ij}^{(r)}$ is the component of the strain tensor **ε**^(*r*)^. As such, the diffraction elastic constant *E*^*hkl*^ for the {*hkl*} grain family is given by $E^{{\it{hkl}}} = \sigma _{\mathrm{a}}/\varepsilon ^{{\it{hkl}}}$. It can be shown that $E_{{\it{hkl}}} = \left[ {(3a - 2b)/3 + 2c + (2b - 2c)(1 - 2\Gamma )} \right]^{ - 1}$, where 3*a*, 2*b* and 2*c* are the parameters that combine both the anisotropic elastic constants of the single-crystal grain and the isotropic elastic constants of the matrix, which will be reported in detail in a follow-up paper; the orientation index parameter Γ is defined as $\Gamma = (h^2k^2 + l^2k^2 + h^2l^2)/(h^2 + k^2 + l^2)$. To obtain the diffraction elastic constants for stainless steel in Table 1, we used the experimental values of single-crystal elastic constants from Clausen et al.^8^, *C*_11_ = 204.6 GPa, *C*_12_ = 137.7 GPa and *C*_44_ = 126.2 GPa, and also calculated the corresponding isotropic elastic constants for polycrystalline stainless steel without texture, i.e., the bulk modulus $\overline K = 160\;\mathrm{GPa}$ and the shear modulus $\bar \mu = 74\;\mathrm{GPa}$. The error bar for *E*^*hkl*^ (Micro) in Table 1 is deterministic (without variation) within the model assumptions adopted.

### CPFE model

A CPFE model is developed to study the nonlinear lattice strain behaviour as well as the impact of Type II and Type III residual stresses on the mechanical behaviour of AM stainless steel. The crystal plasticity constitutive equations are formulated within the rate-dependent, finite-strain framework of elastic–plastic deformation for individual grain crystals^35^. Here we present the major constitutive equations and highlight the new development that accounts for Type II and Type III residual stresses in AM stainless steel. Within each single-crystal grain, the deformation gradient tensor **F** is given in terms of the elastic deformation gradient tensor **F**^*e*^ and plastic deformation gradient tensor **F**^*p*^ using the multiplicative decomposition, **F** = **F**^*e*^ **F**^*p*^. The second Piola–Kirchhoff stress T^*^ is given by

1 $${\bf{T}}^ \ast = {\bf{L}}^{(r)}({\bf{E}}^e - {\bf{E}}^ \ast )$$

In Eq. (1), **L**^(*r*)^ is the elastic stiffness tensor of a single-crystal grain as defined in the micromechanics model; **E**^*e*^ is the elastic Green strain tensor given by

2 $${\mathbf{E}}^e = 1/2\left( {{\mathbf{F}}^{e{\mathrm{T}}}{\mathbf{F}}^e - {\mathbf{I}}} \right)$$

where **I** is the second-rank identity tensor and E^*^ is the eigen-strain tensor that reflects the residual lattice strain measured by in situ SXRD before loading and thus captures the effect of the Type II intergranular residual stress. The rate of change of **F**^*p*^ is given by **F**^*p*^ = **L**^*p*^ **F**^*p*^ and ${\mathbf{L}}^p = \mathop {\sum}\nolimits_{i = 1}^{12} {\dot {\gamma} _i^p{\mathbf{m}}_i \otimes {\mathbf{n}}_i}$, where $\dot {\gamma} _i^p$ is the plastic shear rate on the *i*-th slip system, **m**_*i*_ and **n**_*i*_ are unit vectors of the associated slip direction and slip plane normal, respectively, and ⊗ denotes the tensor product between the two vectors. For austenitic stainless steel with the face-centred cubic structure, 12 $\left\{ {111} \right\}\left\langle {110} \right\rangle$ slip systems are considered. The plastic shearing rate $\dot {\gamma} _i^p$ is given by a power law

3 $$\dot {\gamma} _i^p = \dot {\gamma} _0^p\left| {\frac{{{\tau} _i - b_i}}{{s_i}}} \right|^{1/m}{\mathrm{sgn}}({\tau} _i - b_i)$$

where *τ*_*i*_ is the resolved shear stress given by $\tau _i \approx {\mathbf{T}}^ \ast :{\mathrm{sym}}({\mathbf{m}}_i \otimes {\mathbf{n}}_i)$, $\dot {\gamma} _0^p$ is the reference plastic shear rate and *m* is the strain rate sensitivity. In Eq. (3), *s*_*i*_ is the slip resistance with an identical initial value of *s*_0_ for all the slip systems and it evolves according to $\dot s_i = \mathop {\sum}\nolimits_j {h_{ij}} \left| {\dot {\gamma} _j^p} \right|$ and $h_{ij} = q_{ij}h_0(1 - s_j/s_s)^a$, where *q*_*ij*_ is the latent-hardening matrix; the diagonal elements of *q*_*ij*_ are 1.0 and off-diagonal elements are 1.4. The hardening parameters *h*_0_, *a* and *s*_*s*_ are taken to be identical for all slip systems. Twinning shear is not accounted for in the crystal plasticity model, as in situ SXRD data in Supplementary Fig. 7 and post-mortem TEM data in Supplementary Fig. 8 indicate that deformation twinning plays a negligible role at low strain levels (<10%)^39^.

Following Hu et al.^14^, we represent the effect of Type III residual stresses in terms of the back-stress tensor **B** that gives rise to the tension–compression asymmetry. The so-called intergranular and intragranular back stresses in previous studies^27,28^ are collectively considered as Type III residual stresses in this work, as both are associated with heterogeneous dislocation distributions within grains. The back-stress tensor **B** is assumed to be deviatoric within the present pressure-independent crystal plasticity model. The rate of change of **B** is taken as the sum of the rate of change of back stress in the 12 {111}110 slip systems that is respectively proportional to the corresponding plastic shear rate, i.e.,

4 $${\dot{\mathbf{B}}} = \mathop {\sum}\limits_{j = 1}^{12} {h_be^{ - k{\gamma} _j^p}\dot {\gamma} _j^p\mathrm{sym}({\mathbf{m}}_j \otimes {\mathbf{n}}_j)}$$

where *h*_*b*_ and *k* are the material constants and taken to be identical for all the slip systems. The back-stress tensor **B** is calculated by time integration of Eq. (4) with the initial value of **B**_0_, giving a nonlinear, rate-independent evolution of **B** with increase of plastic shear strain, which is extension of a similar scalar relationship between the back stress and plastic strain by Pham et al.^28^. Then the back stress on the *i*-th slip system *b*_*i*_ is calculated by resolving **B** back onto individual slip systems, i.e.,

5 $$b_i = {\mathbf{B}}:{\mathrm{sym}}({\mathbf{m}}_i \otimes {\mathbf{n}}_i)$$

The above constitutive model was implemented in the finite element simulation package ABAQUS/Explicit^36^ by writing a user material subroutine VUMAT. The parameters of the crystal plasticity model used are listed in Supplementary Table 2, whereas the evaluation of E* and **B**_0_ will be discussed in detail later. In finite element simulations, we constructed a three-dimensional cubic polycrystalline structure with 8000 cubic elements. Each element represents one single-crystal grain with an assigned orientation based on the EBSD data measured from the AM stainless steel sample. During each CPFE simulation, the finite element polycrystal structure was first relaxed prior to loading and then subjected to uniaxial tensile or compressive deformation with the strain rate of 0.001 s^−1^. The lattice strain in different grain families was calculated from the elastic Green strain tensor **E**^*e*^ resolved in LD or TD. The lattice strain for each grain family was determined by averaging the elastic strain of grains within a deviation of ±5° from the scattering vector direction with respect to LD or TD.

We stress that E* is the initial eigen-strain tensor arising from type II intergranular internal stresses and **B**_0_ is the initial back-stress tensor arising from type III intragranular internal stresses. The grain-specific eigen-strain tensor E* was evaluated as follows. Based on the SXRD data of residual lattice strains in the {200}, {311}, {220} and {111} grain families, we assigned the initially estimated values to the eigen-strain tensor E* (i.e., normal components) for individual grains in the four grain families. Then we performed a CPFE simulation to relax the entire polycrystalline aggregate of 8000 grains without loading, so as to obtain the eigen-strain tensor E* for all grains in the simulated polycrystalline aggregate. More specifically, let us consider, as an example, one grain that belongs to both the {220} family along the LD (within a deviation of ±5°) and the {200} grain family along the TD (within a deviation of ±5°). The corresponding E* for this grain is given by

6 $${\mathbf{E}}^ \ast = {\it{\varepsilon }}_{220}^{\mathrm{L}}{\bf{n}}_{220} \otimes {\bf{n}}_{220} + {\it{\varepsilon }}_{002}^{\mathrm{T}}{\bf{n}}_{002} \otimes {\bf{n}}_{002}$$

where ${\it{\varepsilon }}_{220}^{\mathrm{L}}$ and ${\it{\varepsilon }}_{002}^{\mathrm{T}}$ denote the lattice strain along the loading and TD, respectively; **n**_220_ and **n**_002_ denote the unit vector along the [220] and [002] direction, respectively. Suppose the LD is along the *x* axis and the TD along the *y* axis. The corresponding matrix components of E^*^ can be written as

7 $${\mathbf{E}}^ \ast = \left( {\begin{array}{*{20}{c}} {{\it{\varepsilon }}_{220}^{\mathrm{L}}} & 0 & 0 \\ 0 & 0 & 0 \\ 0 & 0 & 0 \end{array}} \right) + \left( {\begin{array}{*{20}{c}} 0 & 0 & 0 \\ 0 & {{\it{\varepsilon }}_{002}^{\mathrm{T}}} & 0 \\ 0 & 0 & 0 \end{array}} \right)$$

For individual grains in the {200}, {311}, {220} and {111} families, the initially estimated values of E^*^ were chosen to be twice the corresponding residual lattice strains measured from the SXRD experiment. After the CPFE relaxation without loading, the residual lattice strains in the {220}, {111}, {200} and {311} grain families were changed from their initially assigned values to closely match the SXRD measurements; meanwhile, the residual lattice strains in other grain families were also obtained. The E* values were not changed during subsequent deformation simulations.

Due to the lack of direct experimental characterization of type III intragranular internal stresses in individual grains, we assigned identical values to **B**_0_ for all grains in the simulated polycrystalline assembly, which approximately represent the average type III intragranular internal stresses in these grains. To match the asymmetry of tensile and compressive yield strengths between experiment and simulation, the initial back-stress components along LD and TD within the build plane are both chosen to be 30 MPa. As such, compared with the annealed sample without the initial back stresses, the compressive yield strength is elevated approximately by 30 MPa, as the initial back stress is directional and effectively increases the resistance to plastic yielding. On the other hand, the tensile yield strength is lowered approximately by 30 MPa, because the initial back stress effectively reduces the resistance to plastic yielding. In addition, the initial back stress component along BD is chosen to be −60 MPa and the shear components of **B**_0_ are zero, so as to make **B**_0_ deviatoric. As a result, such initial back stresses lead to the asymmetry of tensile and compressive yield strengths by ~60 MPa in CPFE simulations. The standard deviation for *E*^*hkl*^ (CPFE) in Table 1 is determined from ten sample calculations.

## Supplementary information

Supplementary Information

## Data Availability

The data supporting the conclusion of this work is available in the main text or in the Supplementary Materials. Raw data are available from corresponding authors upon reasonable request.

## Code availability

The user material subroutine for numerical simulation is available upon reasonable request.
