# Author Correction: Microscale residual stresses in additively manufactured stainless steel

**DOI:** 10.1038/s41467-021-27068-z

**Published:** 2021-11-16

**Authors:** Wen Chen, Thomas Voisin, Yin Zhang, Jean-Baptiste Forien, Christopher M. Spadaccini, David L. McDowell, Ting Zhu, Y. Morris Wang

**Affiliations:** 1 Lawrence Livermore National Laboratory, Livermore, CA 94550 USA; 2 Woodruff School of Mechanical Engineering, Georgia Institute of Technology, Atlanta, GA 30332 USA; 3 Present Address: Department of Mechanical and Industrial Engineering, University of Massachusetts, Amherst, MA 01003 USA

**Keywords:** Mechanical properties, Metals and alloys, Theory and computation

Correction to: *Nature Communications* 10.1038/s41467-019-12265-8, published online 25 September 2019.

In this article, the author name Jean-Baptiste Forien was incorrectly written as Jean-Baptiste Florien. The original article has been corrected.

