# Supplementary material for: Microscale residual stresses in additively manufactured stainless steel
Source: Nat Commun. 2019 Sep 25;10:4338. doi: 10.1038/s41467-019-12265-8 (PMC6761200; doi:10.1038/s41467-019-12265-8)
Supplement: Supplementary file 1 — Supplementary Information [file 41467_2019_12265_MOESM1_ESM.pdf]

Supplementary Information

Microscale residual stresses in additively manufactured stainless steel

Wen Chen[1¶], Thomas Voisin[1¶], Yin Zhang[2¶], Jean-Baptiste Florien[1], Christopher M. Spadaccini[1], David L. McDowell[2], Ting Zhu[2*], Y. Morris Wang[1*]

[1]Lawrence Livermore National Laboratory, Livermore, CA 94550, USA

[2]Woodruff School of Mechanical Engineering, Georgia Institute of Technology, Atlanta, GA 30332, USA

[*]Emails: (T.Z.) ting.zhu@me.gatech.edu; (Y.M.W.) ymwang@llnl.gov

[¶]These authors contribute equally to this work.

This Supplementary Information contains Supplementary Tables 1-2 and Supplementary Figures 1-8.

Supplementary Table 1. Laser powder-bed-fusion (L-PBF) processing parameters used for sample fabrication. An open architecture Fraunhofer L37 laser machine was used (see Methods in the main text).

	**Parameter**
Laser spot size (μm)	207
Laser power (W)	330
Scan strategy	Continuous
Scan speed (mm/s)	150
Build layer thickness (μm)	50
Hatching angle (°)	45

Supplementary Table 2. Parameters used for crystal plasticity finite element simulations.

Parameter	Value
$C_{11}\,(\mathrm{GPa})$	204.6
$C_{12}\,(\mathrm{GPa})$	137.7
$C_{44}\,(\mathrm{GPa})$	126.2
$\dot{\gamma}_0\,(\mathrm{s}^{-1})$	0.001
m	0.023
$s_0\,(\mathrm{MPa})$	200
$h_0\,(\mathrm{MPa})$	250
$s_s\,(\mathrm{MPa})$	447
a	0.7
$h_b\,(\mathrm{MPa})$	126.2
k	3000

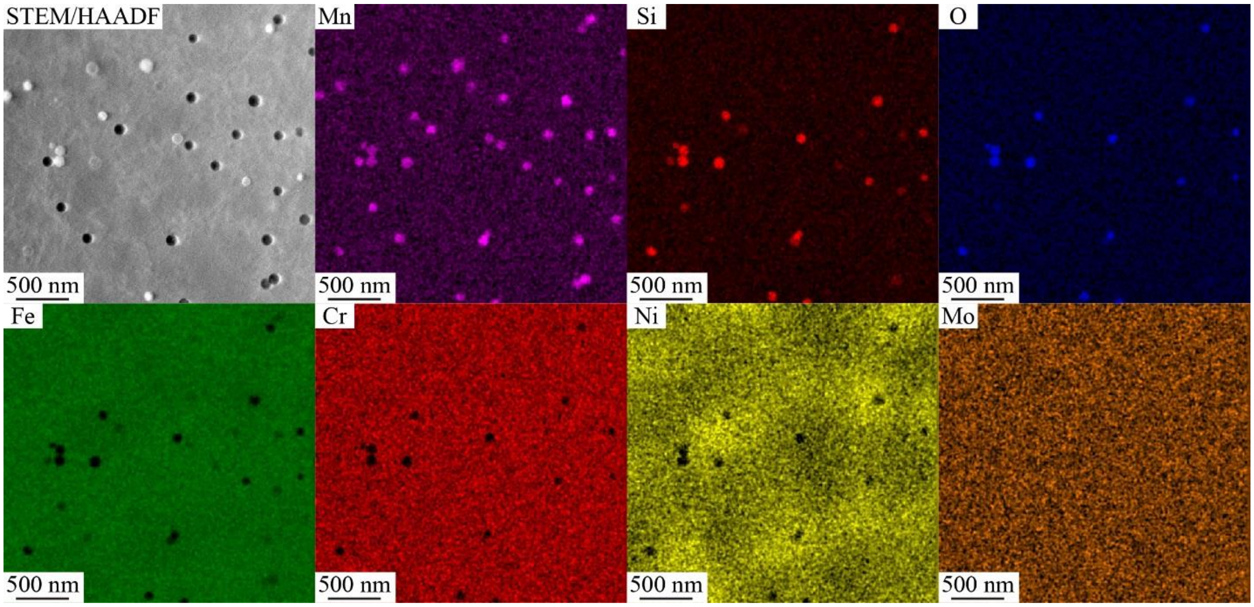

Supplementary Figure 1. Elemental map of the as-printed 316L stainless steel sample. No clear chemical segregation was observed.

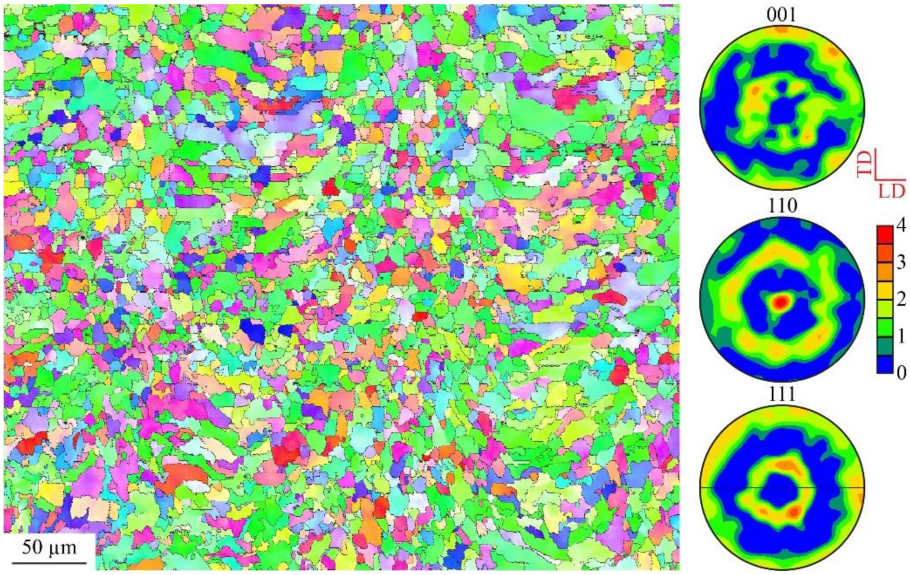

Supplementary Figure 2. Electron backscatter diffraction (EBSD) inverse pole figure and the corresponding pole figures of the annealed sample (500 ˚C for 4 hours). The build direction (BD) is out of plane. TD and LD stand for the transverse and longitudinal directions, respectively.

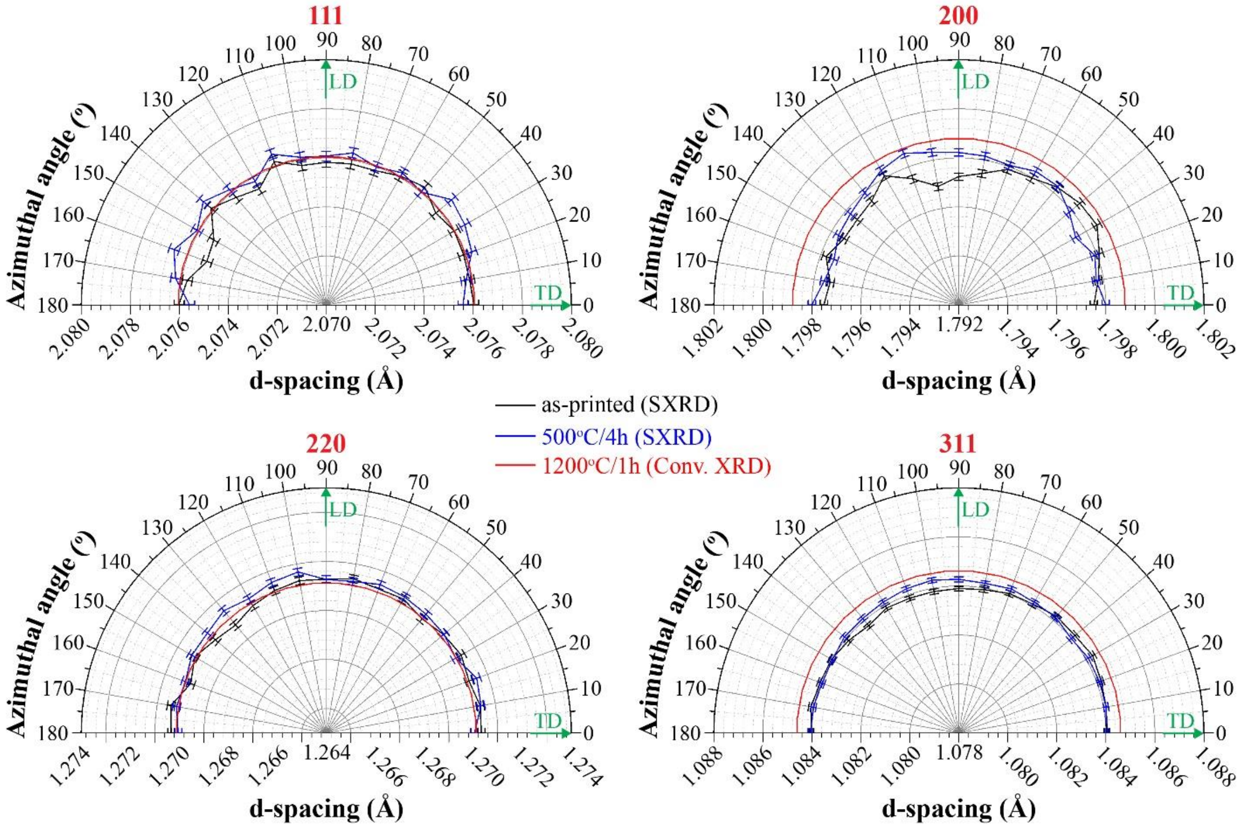

Supplementary Figure 3. Lattice spacing (d-spacing) as a function of the azimuthal angle for three types of samples (as-printed, annealed at 500 ˚C and 1200 ˚C, respectively), shown over 180-angles as a result of the average of equal opposite diffraction. The diffraction data for the reference sample (1200 ˚C-annealed) were obtained by conventional X-ray diffractometry (Conv. XRD) due to its large grain size. The error bars on the synchrotron XRD (SXRD) data come from the estimated error due to sample-detector distance variations as we change samples between measurements (see Methods in the main text). Note that 200 and 311 reflections contain the relatively larger residual strains due to their lower stiffnesses.

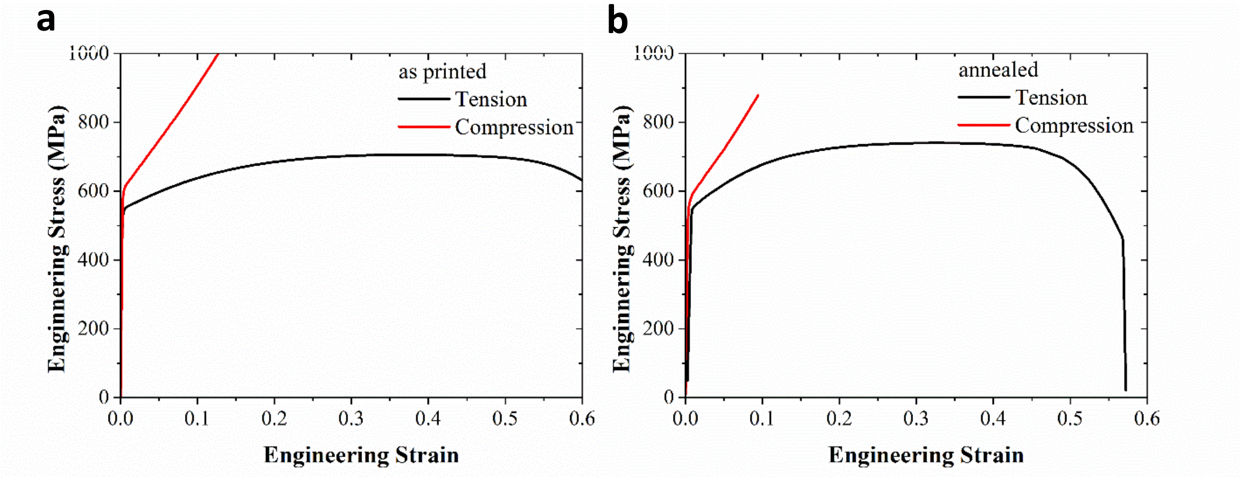

Supplementary Figure 4. Engineering stress-strain curves of tested samples. a As-printed; **b** Annealed (500 °C, 4hrs). Compression was stopped at ~10% strain before the load cell limit was reached.

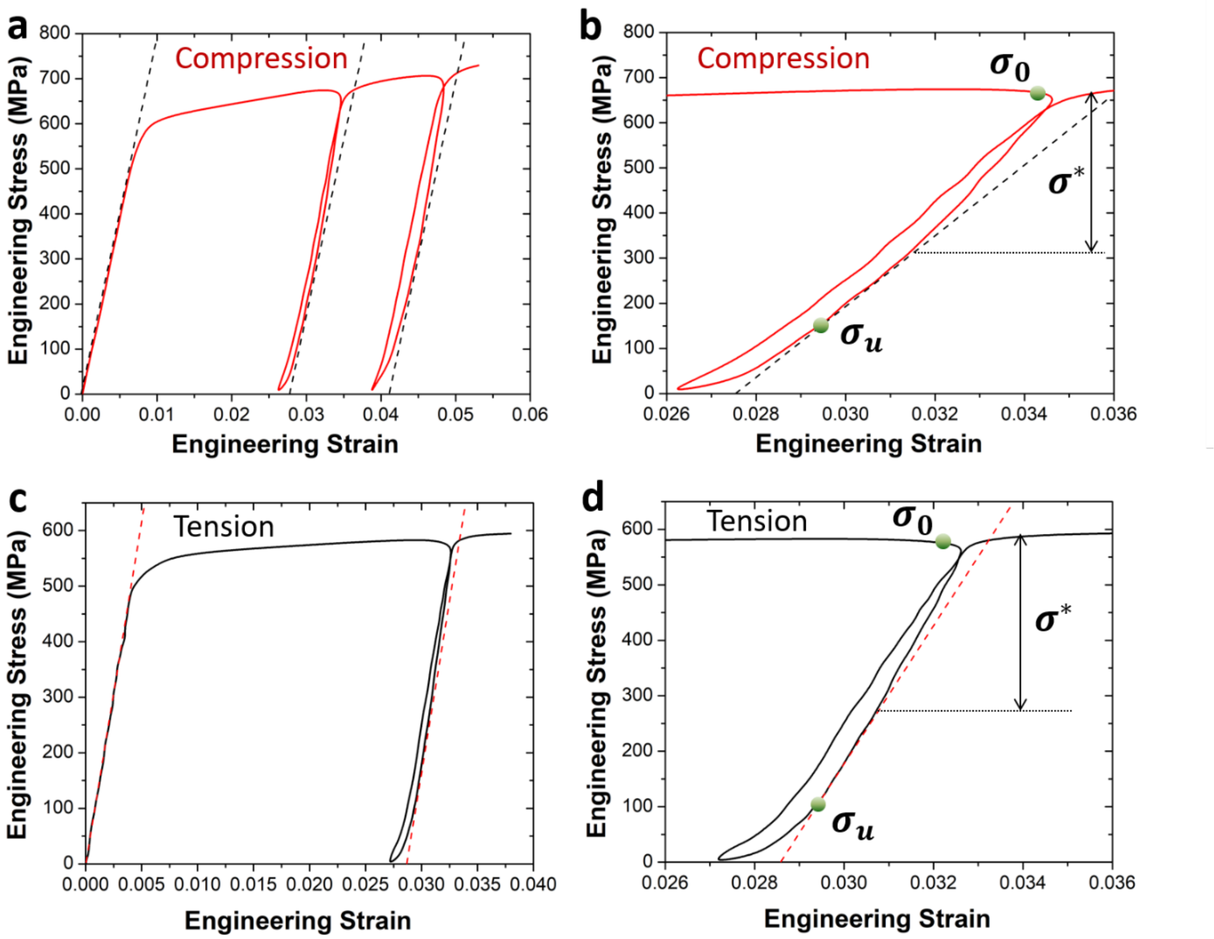

Supplementary Figure 5. Measurements of back stress in the as-printed sample by loading-unloading at ~3% strain under a, b compression and c, d tension. **b** and **d** show the magnified loading- unloading curves at the strain of ~3% in **a** and **c**, respectively. The back stress is calculated with Dickson's method[1]. Namely, the back stress, σ_b, is given by $\sigma_b = \frac{\sigma_0+\sigma_u}{2} - \frac{\sigma^*}{2}$, where σ_0 is the flow stress prior to unloading, σ^* is the effective stress, and σ_u is the reverse yield stress.

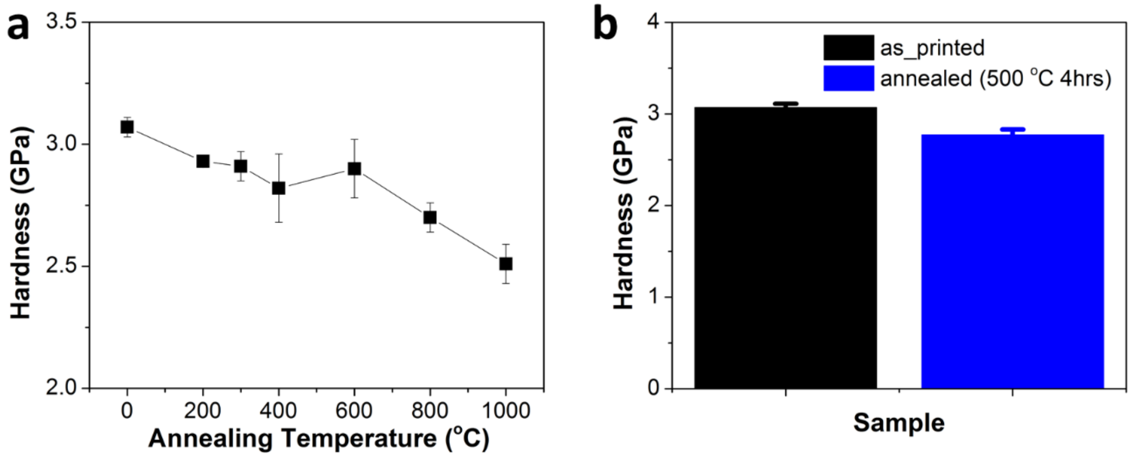

Supplementary Figure 6. Effect of annealing at different temperatures on the nano-hardness of AM 316 stainless steel. a A marginal change of hardness is measured after annealing at temperatures below ~ 600 ˚C for 1 hr, suggesting little microstructure change after annealing at these temperatures (Supplementary Fig. 2). In contrast, nano-hardness reduction is substantial after annealing at temperatures above ~ 600 ˚C. **b** To focus on the residual stress effect on the mechanical behaviour and decouple the microstructural effect, we conducted stress-relief annealing at 500 ^{o}C for 4 hrs. The hardness measurement confirms marginal change compared to the as-printed sample. The error bars in the figures are averaged from at least nine measurements for each sample.

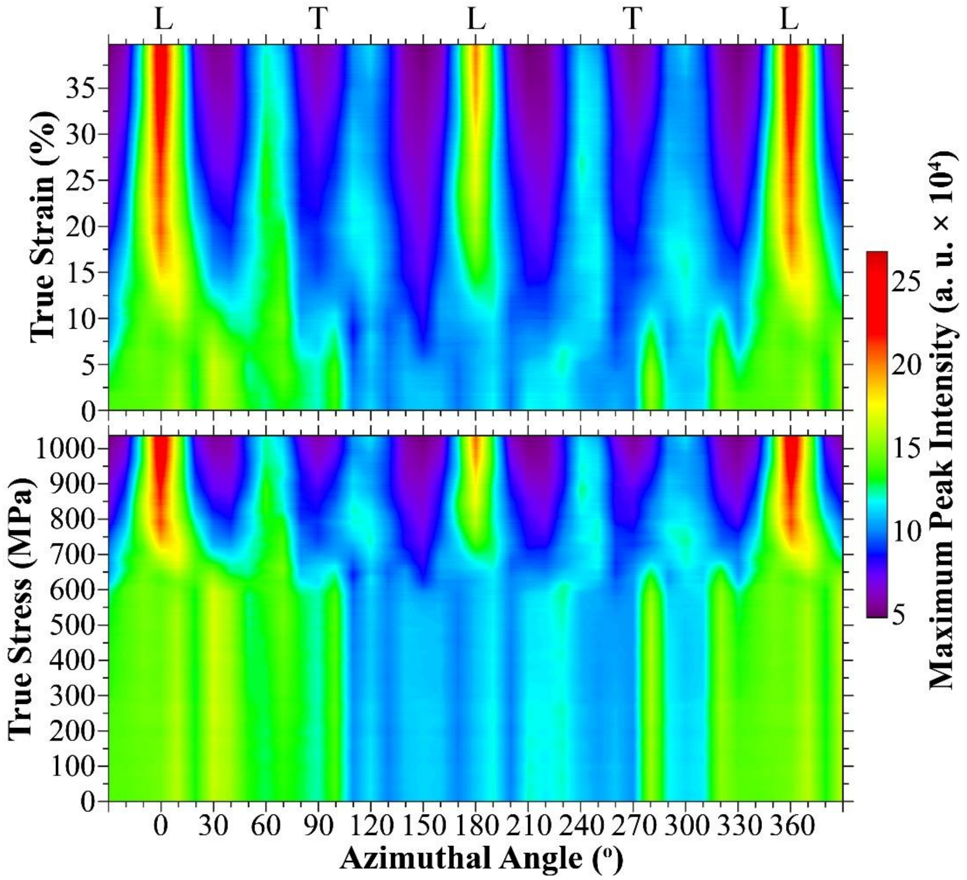

Supplementary Figure 7. Texture evolution during *in situ* tensile testing synchrotron X-ray diffraction (SXRD). Maximum peak intensity distribution around the entire diffracted ring corresponds to the 111 reflection as a function of true strain and true stress for the as-printed material. L and T represent loading and transverse directions, respectively. Note that significant text change is not observed along the loading direction until the strain reaches >10% or the true stress is above ~650-700 MPa, suggesting that twinning does not play a role at low strain levels.

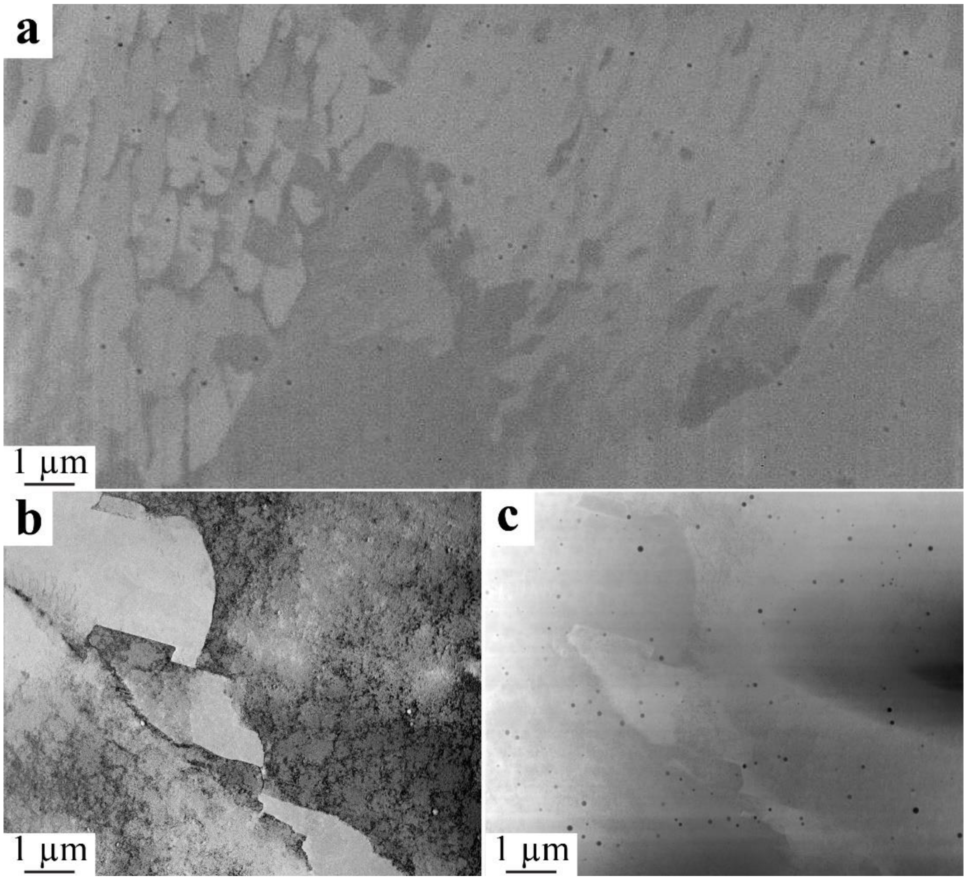

Supplementary Figure 8. Microstructure of the as-printed 316L stainless steel after ~1% tensile strain. a A secondary electron scanning electron micrograph (SEM) in a large area shows no evidence of deformation twinning. **b** and **c** TEM bright field and STEM/HAADF images, respectively, reveal no evidence of deformation twinning.

Supplementary Reference

1 Dickson, J., Boutin, J. & Handfield, L. A comparison of two simple methods for measuring cyclic internal and effective stresses. *Mater. Sci. Eng.* **64**, L7-L11 (1984).
